# Atypical working hours are associated with tobacco, cannabis and alcohol use

**DOI:** 10.1093/eurpub/ckac131.256

**Published:** 2022-10-25

**Authors:** N Hamieh, G Airagnes, A Descatha, M Goldberg, F Limosin, Y Roquelaure, C Lemogne, M Zins, J Matta

**Affiliations:** 1 UMS11, INSERM, Villejuif, France; 2 Faculty of Health, Université Paris Cité, Paris, France; 3 DMU Psychiatrie et Addictologie, AP-HP, Centre Université de Paris, Paris, France; 4 Poison Control Center, Academic Hospital CHU Angers, Angers, France; 5 UMR_S 1085, INSERM, Angers, France; 6 Department of Occupational Medicine, Donald and Barbara Zucker School of Medicine, Hofstra/Northwell, USA; 7 U1266, INSERM, Paris, France; 8 DMU Psychiatrie et Addictologie, Hôpital Corentin-Celton, Issy-Les-Moulineaux, France; 9 Consultations de Pathologie Professionnelle, University of Angers, Angers, France

## Abstract

**Background:**

We examined prospective associations between atypical working hours, substance use and sugar and fat consumption.

**Methods:**

In the French population-based CONSTANCES cohort, 47,288 men and 53,324 women currently employed included between 2012 and 2017 were annually followed for tobacco and cannabis use; among them, 35,647 men and 39,767 women included between 2012 and 2016 were also followed for alcohol and sugar and fat consumption. Three indicators of atypical working hours were self-reported at baseline: working at night, weekend work and non-fixed working hours. Generalized linear models computed odds of substance use and sugar and fat consumption at follow-up according to atypical working hours at baseline while adjusting for sociodemographic factors, depression and baseline substance use if appropriate.

**Results:**

Working at night was associated with increased tobacco use in women [odds ratios ranging from 1.45 to 1.48], with increased cannabis use in men [from 1.40 to 1.54] and with increased alcohol use in both men and women [from 1.12 to 1.14]. Weekend work and non-fixed working hours were associated with increased tobacco and alcohol use in both men and women [from 1.15 to 1.54 and 1.12 to 1.14, respectively]. Dose-dependent relationships were found regarding the association between working at night and tobacco use in women as well as regarding non-fixed working hours and tobacco use in both men and women (P for trends <0.001).

**Conclusions:**

The potential role of atypical working hours on substance use should be considered by public health policy makers and clinicians in information and prevention strategies.

**Key messages:**

• Night shifts were associated with increased smoking in women with dose-dependent relationships, cannabis use in men and alcohol use in both.

• Weekend work and non-fixed working hours were associated with increased alcohol and tobacco use with dose-dependent relationships in men and women.

